# *Acacia hydaspica* ethyl acetate extract protects against cisplatin-induced DNA damage, oxidative stress and testicular injuries in adult male rats

**DOI:** 10.1186/s12885-017-3898-9

**Published:** 2017-12-21

**Authors:** Tayyaba Afsar, Suhail Razak, Muhammad Rashid khan, Ali Almajwal

**Affiliations:** 1 Department of Biochemistry, Quaid-i-Azam University, Islamabad, Pakistan; 2 Department of Animal Sciences, Quaid-i-Azam University, Islamabad, Pakistan; 3 Department of Community Health Sciences, College of Applied Medical Sciences, King Saud University, Riyadh, KSA Saudi Arabia

**Keywords:** Cisplatin, Testicular impairment, Antioxidant enzymes, Reproductive hormones, DNA damages

## Abstract

**Background:**

Cisplatin (CP), an effective anticancer agent, carries the risk of impairing testicular function leading to infertility. The present study aimed at evaluating the protective effect of *A. hydaspica* ethyl acetate extract (AHE) against CP-induced oxidative stress and testicular injuries in rats.

**Methods:**

Rats were divided into six groups (*n* = 6). Group I (control), group II (CP single dose on day 16). Group III received AHE for 21 days. Group IV (CP + AHE; post- treatment group). Group V (AHE + CP; pre-treatment group) and group VI (CP + Sily).

**Results:**

CP treatment reduced serum testosterone (T), LH and FSH, decreased the activity level of antioxidant enzymes while increased the concentration of oxidative stress markers, i.e. thiobarbituric acid reactive substances (TBARS), H_2_O_2_ and nitric oxide (NO) along with corresponding DNA damages. Furthermore, CP induced adverse morphological changes in testis of rats including reduced epithelial height and tubular diameter, increased luminal diameter with impaired spermatogenesis. Pre and post-treatment with AHE reduced the side effects of CP in testis tissues through improvement in the reproductive hormonal secretions, enzymatic activities, histological and DNA damage parameters. Pretreatment seems to be more effective and equivalent to silymarin group in reversing the CP deleterious effects as compared to post-treatment.

**Conclusion:**

The results demonstrated that *A. hydaspica* treatment in CP-induced testicular toxicity augments the antioxidants defense mechanism, reverted the level of fertility hormones, suppressed the histomorphological alterations and DNA damages and thus provides the evidence that it may have a therapeutic role in free radical mediated diseases.

## Background

Cisplatin (CP) is a platinum-derived anti-neoplastic, DNA alkylating agent used widely as a front line adjuvant therapy against various cancers such as testicular, gut, stomach, head, neck, ovarian, cervical, germ cell tumors and non-small cell lung carcinoma [1]. Use of CP in the treatment of testicular cancer, even at the progressive stage of the disease has promising results, however, unwanted secondary effects are associated with its use causing reduction of testicular weight, azoospermia and transient or persistent demolition of male reproductive proficiency [2–4]. Spermatogenesis is an intricate phenomenon which is greatly inveigle by the hormonal level and environmental conditions most notably the temperature. This phenomenon implicates multitude of testicular cells such as sertoli cells, Leydig cells and peritubular cells [5, 6]. Synthesis of nucleic acid in germ cells especially in spermatogonia is impeded by CP treatment on account of its alkylating abilities [7–9]. Damages induced with CP to Leydig cells resulted in inhibition of testosterone secretion [10].

Pathogenesis of reproductive impairments ensuing to CP exposure is usually attributed by the oxidative stress expedited in deterioration of antioxidant defense system. Within the human body protection against oxidative stress is achieved by self-defense enzymes that catalytically remove the free radicals and other reactive species. These include; superoxide dismutase, catalase and glutathione peroxidase [11], whereas CP decreases the functioning of these enzymes, thereby inducing oxidative damages in testicular tissue [12, 13]. Current chemotherapeutic agents are mostly non-selective in their efficacy and kill dividing cells rapidly including cancer, normal and stem cells. Such injurious agents also effect spermatogenic cells and fallouts in men infertility [14]. There are cumulative evidences proposing that utilization of antioxidants could be persuasive in ameliorating cisplatin-prompted toxicity [15–17]. Hence there is a necessity to develop combinatorial therapies that will reduce the CP resistance and minimizing its side effects. Plants possess a wide range of constituents which are capable of preventing various oxidative stress related ailments, including male reproductive disorders [18], hence frequently administered in recipes with chemotherapeutic drugs to give enhanced protection against their side effects [19].

Various species from the genus *Acacia* exhibit outstanding antioxidant and chemo-preventive possessions that aid in inhibition and treatment of various oxidative stress related ailments [20–22]. *Acacia hydaspica* R. Parker; synonym *A. eburnea* belongs to family Leguminosae [23]. *A. hydaspica* possesses antioxidant, anticancer, anti-hemolytic, anti-inflammatory, antipyretic and analgesic potentials; these activities attributed to the presence of various active secondary metabolites i.e. gallic acid, catechin, rutin, caffeic acid, 7-*O*-galloyl catechin, +catechin and methyl gallate [24]. Polyphenolic compounds isolated from *A. hydaspica* induce apoptosis and inhibit various pro-survival signaling pathways in breast and prostate cancer cell lines, indicating their potential in molecular target based adjuvant chemotherapy [25]. Ethyl acetate extract of *A. hydaspica* (AHE) was selected for the in vivo investigation due to its significant antioxidant capacity [26], and the presence of catechin and gallic acid as chief component, substantial total phenolic and flavonoid content (Table 1).

**Table 1:** TPC, TFC, and chemical constituents of *A. hydaspica* ethyl acetate extract (AHE)

Analysis (AHE fraction)	Observations	(References)
TPC (mg gallic acid equivalent/g dry sample)	120.3 ± 1.15	[25]
TFC (mg rutin equivalent/g dry sample)	129 ± 1.32	[25]
HPLC-DAD (Identification of compounds using standard polyphenolics)	i. Gallic acid (275 nm, RT: 4.516, 52.92 μg/100 mg dry powder) ii. Catechin (279 nm, RT 11.427, 8648 μg/100 mg dry powder) iii. Myricetin (368 nm, RT: 17.082, 34.60 μg/100 mg dry powder)	[25]
Purified compounds (identified by NMR characterization of compounds)	i. 7-*O*-galloyl catechin (GC)(187.5 mg/g) ii. Catechin (C), (100 mg/g) iii. Methyl gallate (MG), (37.5 mg/g)	[24, 27]

*TPC* Total Phenolic content, *TFC* Total flavonoid content. Information derived from previous lab investigations

Previous researches indicated that catechins possess persuasive antioxidant, anti-inflammatory, immunomodulatory, and anticancer potential [27]. Similarly epigallocatechin gallate (EG) provided significant protection against testicular toxicity and spermiotoxicity in rats exposed to cisplatin. This is attributed to the beneficial properties of EG, such as inhibition of oxidant/nitrative stress, inflammation, and apoptosis [28]. Silymarin, a natural polyphenolic flavonoid, is extracted from the seeds of *Silybum marianum*. This flavonoid is known as a potent antioxidant which exerts its protective role in oxidative stress induced disorders [29, 30]. Moreover,silymarin treatment afford protection against antineoplastic drug induced testicular toxicity by maintaining sperm count, testicular spermatid head concentration, daily sperm production, serum testosterone levels and normal spermatogenesis [31]. Therefore, it has become requisite to supplement with antioxidants to minimize the toxicity caused by anti-cancer medications.

Based on earlier research on the protective efficacy of polyphenolic compounds against drug induce reproductive toxicity in animal models and antioxidant properties of *A. hydaspica*; current experiment was designed to determine the potential of ethyl-acetate extract of *A. hydaspica* to attenuate CP-induced testicular toxicity and oxidative stress in rats. Silymarin was choisen as standard plant derived protective agent for reference. Comet assay and the activity level of various antioxidant enzymes of testicular tissues, histopathological evaluation along with biochemical and hormone analysis of serum was performed to evaluate the protective potential of *A. hydaspica* against cisplatin induced testicular damages.

## Methods

### Plant collection

The aerial parts (bark, twigs, and leaves) of *A. hydaspica* were collected from Kirpa charah area Islamabad, Pakistan. Plant specimen was identified by Dr. Sumaira Sahreen (curator at Herbarium of Pakistan, Museum of Natural History, Islamabad). A voucher specimen with Accession No. 0642531 was deposited at the Herbarium of Pakistan, Museum of Natural History, Islamabad for future reference.

### Drug and plant dose preparation

Cisplatin (CP) injection (Sigma-Aldrich, St. Louis, MO, U.S.A.) was dissolved in saline and 7.5 mg/kg body weight dose of CP was selected on the basis of previous literature to induce testicular toxicity [32]. *A. hydaspica* methanol extract was fractionated as previously described [33], and its ethyl acetate extract (AHE) (the most bioactive extract under in vitro examinations and containing bioactive polyphenols [34] was selected for further in vivo investigation. Silymarin (100 mg/kg b.w) and AHE (400 mg/kg b.w) were freshly prepared in distilled water before dosing. The dose of extract was selected based on our pilot experiment.

### Animals

Male Sprague Dawley rats (200–225 g) were kept in the Primate Facility at Quaid-i-Azam University, Islamabad. The animals were placed at room temperature, fed with standard pellet diet and tap water under 12 h light/dark cycle at 25 ± 3 °C. Guidelines of national institute of animal health (NIH guidelines) were strictly adapted for experimentations. The ethical board of Quaid-i-Azam University, Islamabad permitted the experimental protocol (Bch#264).

### Acute toxicity evaluation

The acute toxicity study was conducted as per the guidelines 425 of Organization for Economic Cooperation and Development (OECD) for testing of chemicals for acute oral toxicity [35]. Male Sprague Dawley rats were kept in fasting conditions for overnight with just water availability. Three animals were orally administered with dose-of 50 mg/kg bw and were monitored for mortality rate for 72 h. No initial progression of toxicity was observed, but the methodology was subsequently followed with augmented amount of oral doses i.e., 100, 200, 400, 1000, 2000, 3000 and 4000 mg/kg bw of AHE, while the control group received saline (10 ml/kg). Three animals were used for each treatment. General behavioral changes were detected by the previously described procedure [36]. Animals were observed continuously for 2 h and parameters which were observed were convulsion, tremor, aggression, excitation, loss of grasp, altered reactivity to touch, and sedation [37]. AHE was found to be safe at all tested doses (up to 4000 mg/kg b.w) and it did not induced any noxious symptom in rats like sedation, convulsions, diarrhea and irritation. 400 mg/kg bw dose was selected for further in vivo evaluation.

### Experimental design

**Fig. 1:**
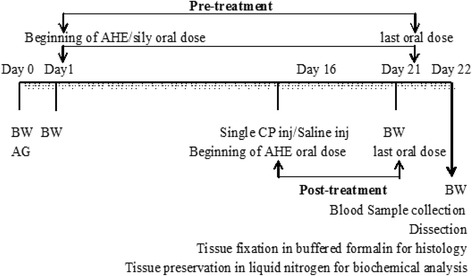
Time line of experimental protocol. BW: body weight, AG: animal grouping, inj: injection, AHE: *A. hydaspica* ethyl-acetate extract, CP: cisplatin, Sily:silymarin

The experimental plan was designed according to previous studies [38, 39] with minor modifications. Figure 1 summarized the experimental plane. Animals were distributed into six groups (*n* = 6), except the CP group (*n* = 10) due to risk of mortality.

The following treatment procedure was adopted for the study.

Group I: Control received water for 21 days, and saline injection (2 ml/kg, i.p) on day 16.

Group II: CP treated; received one dose of CP (7.5 mg/kg b.w., i.p.) on day 16th of experiment for inducing organ toxicity, and distilled water for 21 days (oral).

Group III: AHE treated; 400 mg/kg body weight/day oral dose for 21 days.

Group IV: CP + AHE (post treated group); CP on day 16 after continuous administration of oral distilled water and AHE (400 mg/kg b.w/day, p.o.) was administered from day 16 to 21.

Group V: AHE + CP (pretreated group); received 400 mg/kg body weight/day, p.o. for 21 days and CP (7.5 mg/kg b.w., i.p.) on day 16.

Group VI: Silymarin + CP; received 100 mg/kg b.w., p.o. dose every other day (11 doses/21 days) and CP (7.5 mg/kg b.w., i.p.) on day 16.

Initial and final body weights of rats were recorded. Rats were secrified by cervical dislocation after final dosing. Trunk blood was taken with 23 G1 syringes and collected in sterile falcon tubes. Blood was centrifuged at 500×g for 15 min at 4 °C to obtained serum and kept at −80 °C until hormonal and biochemical analysis**.** After taking blood, testis were dissected out and weigh after washing in saline. Right testicular tissue was stored at -80 °C for tissue homogenate tests, while left testes was stored in 10% phosphate buffered formalin for histological processing. Testicular tissues (100 mg) of each tissue sample was homogenized in 10 volume of 100 mM KH_2_PO_4_ buffer containing 1 mM EDTA, pH 7.4 and centrifuged at 12000×g for 30 min at 4 °C. The supernatant was collected and used for the determination of various biochemical markers.

### Hormone analysis

Testosterone concentration in the serum sample of experimental groups was determined through Astra Biotech kit, Immunotech Company. Concentration of LH and FSH in the serum of different treatment groups were estimated through GenWay Biotech, Inc. Immunoassay Test Kits.

### Protein estimation

The total protein content of homogenate was determined by total protein kit (AMP Diagnostics, Austria) using bovine serum albumin as a standard.

### Biochemical analysis

#### Measurement of tissue antioxidant status

Catalase (CAT) and Peroxidase (POD) activity was determined by the protocol of Khan et al. with slight modifications [40]. Kakkar et al. method was utilized for the assessment of superoxide dismutase (SOD) activity [41]. The Quinone reductase (QR) activity in tissues of different treatment groups was evaluated as described by Benson and colleagues [42]. Reduced glutathione (GSH) activity was checked as described by protocol of Jollow [43]. Scheme of Habig et al. [44] was followed for the estimation of Glutathione-S-transferase (GST) potency. Glutathione reductase (GR) activity in tissue samples was analyzed as described by Carlberg and Mannervik [45]. Glutathione peroxidase (GPx) activity was assessed as described by Mohandas and coworkers [46]. The activity of γ-glutamyl transpeptidase (γ-GT) was checked following Orlowski et al. scheme [47].

#### Measurement of oxidative stress markers in testis

Estimation of hydrogen peroxide activity in tissue samples was monitored by the method described earlier [48]. For the execution of nitrite assay, Griess reagent was utilized [49]. Protocol of Iqbal et al. [50] was adopted with slight modifications for the assessment of lipid peroxidation (TBARS/LPO).

### DNA damage analysis

Protective effect of *A. hydaspica* AHE fraction on CP persuaded testicular DNA damage was assessed by comet assay. Single cell gel electrophoresis (Comet assay) was performed by following the protocol of Dhawan et al. [16] to assess the DNA damage in testicular tissues. CASP 1.2.3.b image analysis software was used to evaluate the extent of DNA damage. In each sample, 50–100 cells were analyzed for following prameters by software

i. Comet length = Length of the entire comet from left border of head area to end of tail (in pixels)
ii. Tail moment = Tail DNA% x Tail Length ([percent of DNA in the tail] x [tail length])
iii. Head DNA = Amount of DNA in the comet head (Sum of intensities of pixels in the head
iv. % Head DNA = Percent of DNA in the comet head
v. Tail DNA = Amount of DNA in the comet tail (Sum of intensities of pixels in the tail)
vi. % Tail DNA = Percent of DNA in the comet tail

### Histopathological examination by light microscopy

For histopathological examination, testicular tissues from each group were fixed in a fixative containing absolute alcohol (85 ml), glacial acetic acid (5 ml) and 40% formaldehyde (10 ml). After dehydration tissue samples were fixed in parafin to prepare blocks for microtomy. Tissues were sectioned 4–5 μm with microtome and stained with Hemotoxilin-Eosin (H&E) and studied under a light microscope (DIALUX 20 EB) at 10X and 40X. Photographs were taken with same the zoom and the camera settings were used and histological parameters were analyzed.

### Morphometry and Planimetry

For morphometric studies, the seminiferous tubule diameter and seminiferous tubule epithelial height of testicular tissue were measured by using Image J software (National Institute of Health, Bethesda, MD, USA) [51]. Shortly, a picture of known distance in micrometer was used for setting scale and conversion of values from pixels to micrometers.

Area of seminiferous tubule and interstitial space was determined by planimetry, using Image J software. The area in μm^2^ was calculated following the method of Islam et al. and Jensen [51, 52]. Briefly, 25 pictures/animal (40X) of known area were selected and the area of seminiferous tubules and interstitial space was determined by the free selection tool of the software. The area percentage (%) was calculated by the formula:

$$ \%\mathrm{AS}=\left[\frac{\mathrm{AS}\times 100}{\mathrm{T}}\right] $$

Where AS is the area covered by seminiferous tubule.

T is the total area of the field.

Percentage of the mean area was analyzed for comparison between the treated and control groups.

### Statistical analysis

Data are expressed mean ± SEM (*n* = 6). One way analysis of variance (ANOVA) followed by Tukey’s test was used for analyzing the statistical differences between different treatment groups using Graph pad prism 5 software. Level of significance was set at *p* < 0.05.

## Results

### Acute toxicity evaluation

Since toxicity of the test sample was a focal concern, hence acute toxicity evaluation was done before proceeding to in vivo experiment. AHE was found to be safe at all tested doses (up to 4000 mg/kg b.w) and it did not induce any noxious symptom in rats like sedation, convulsions, diarrhea and irritation. During the 3 days of the assessment, no mortality was observed. Therefore, one tenth of the maximum dose, 400 mg/kg b.w. was used for in vivo evaluation of AHE.

### AHE treatment is not associated with toxicity

**Fig. 2:**
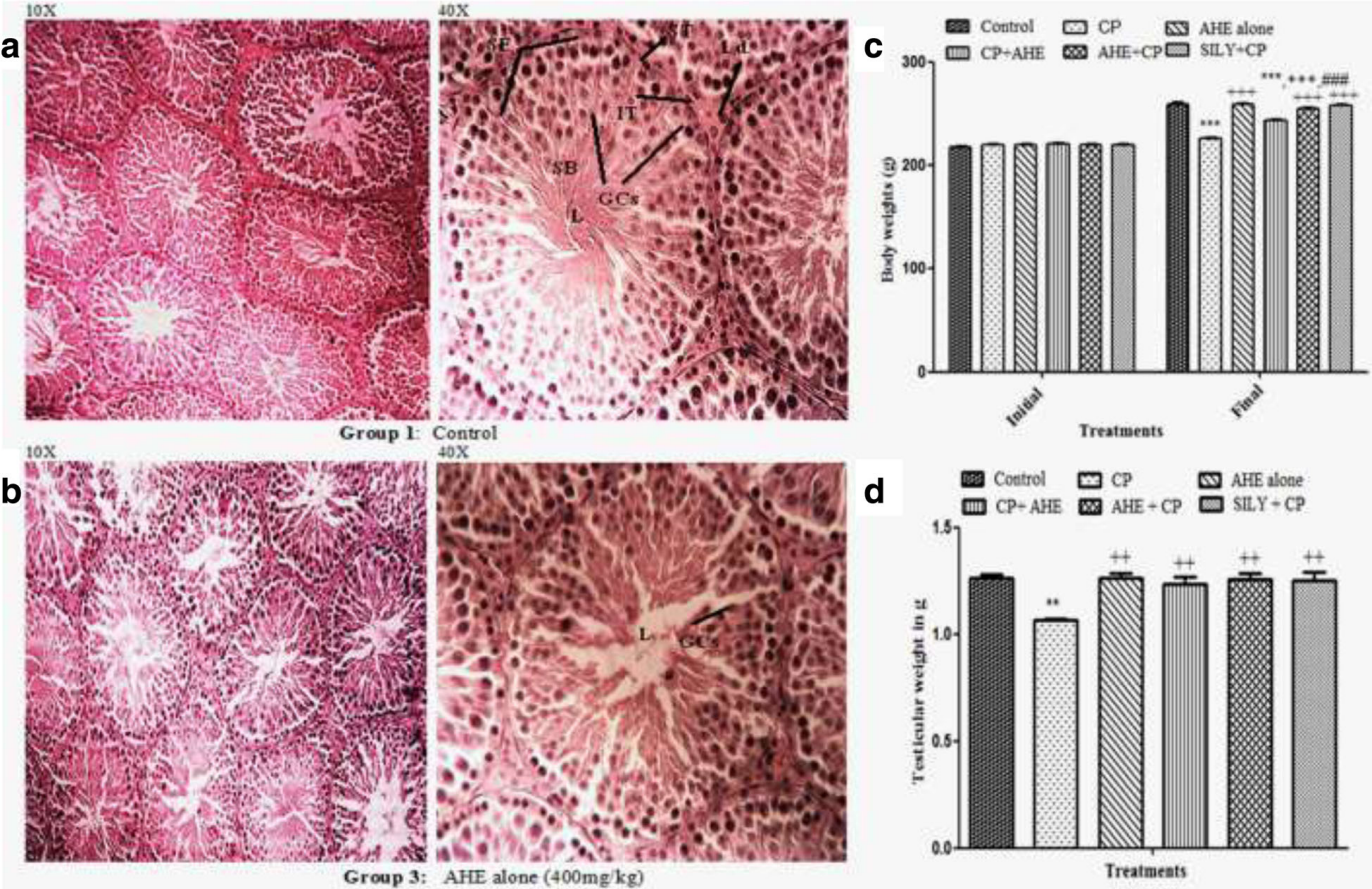
Histopathological comparison of control and AHE group (**a**, **b**). Initial and final body weights of different treatment groups (**c**). Testicular weights of various treatment groups (**d**). Statistical difference in final body weight and testicular weight of different treatment groups were analyzed by one way ANOVA followed by Tukeys Multiple Comparison Tests

In all treatment groups no clinical signs of toxicity (such as, unusual salivation, flick-king movements, shiver, head and forelimb clonuses, spasms, incoordination, diarrhea and increased diuresis) were observed. No death was witnessed in any treatment group during the experimental period. The testicular tissues from either control or AHE treated rats revealed no obvious variations in histo-architecture (Fig. 2a and b). The animals treated with AHE showed similar body weight gain compared to the control group (Fig. 2c). Similarly, no significant change in testicular weight was recorded in AHE and control groups (Fig. 2d), suggesting that there was no toxicity associated with AHE dosage. The results strongly suggest the protective effect of AHE treatment with no adverse effects associated with treatment.

### Effect of AHE treatment on male reproductive hormones of rat

Reproductive hormones such as testosterone (T), luteinizing hormone (LH) and follicle stimulating hormone (FSH) are considered as imperative serological biomarkers to estimate testicular toxicity. The serological concentration of T, LH and FSH are highly susceptible to oxidative stress induced by CP as shown in Table 2, indicating that CP administration altered the secretion of pituitary and gonadal hormones. CP inoculation significantly (*p* < 0.0001) decreased the concentration of serum T, LH and FSH which was ameliorated significantly (*p* < 0.0001) by oral administration of AHE compared to CP group, however the hormonal concentration did not restore to normalized control values. However, AHE alone showed the same serum hormone concentration like that of control group. AHE + CP treatment was more effective (*p* < 0.0001) as compared to CP + AHE in preventing CP intoxication signifying the protective role of AHE. AHE + CP exhibited analogous effect on serum reproductive hormone concentration as a CP + silymarin treated group.

**Table 2:** Effect of cisplatin (CP) and different treatments of AHE on male reproductive hormones

Group	T (ng/ml)	LH (mIU/ml)	FSH (mIU/ml)
Control	4.55 ± 0.09	2.30 ± 0.04	1.46 ± 0.012
CP	2.32 ± 0.06^***^	1.32 ± 0.02^***^	0.79 ± 0.035^***^
AHE alone	4.59 ± 0.06^+++^	2.31 ± 0.04^+++^	1.46 ± 0.013^+++^
CP + AHE	2.85 ± 0.09^***,+++,###^	1.46 ± 0.02^***,+,###^	1.00 ± 0.053^***,+++,###^
AHE + CP	4.20 ± 0.07^*,+++^	2.09 ± 0.06^*,+++^	1.32 ± 0.022^*,+++^
CP + Sily	4.16 ± 0.04^*,+++^	2.08 ± 0.06^**,+++^	1.31 ± 0.013^**,+++^

*n* = 6/group; values are expressed as mean ± SEM; ^*, **, ***^ indicate significance from the control group as mean ± SEM at *p* < 0.05, *p* < 0.001 and *p* < 0.0001 probability level, respectively; ^++, +++^ indicate significance from the CP group at *p* < 0.001 and *p* < 0.0001 probability level, respectively; ### indicates comparison of AHE + CP pre-treated group vs. CP + AHE post-treated group at *p* < 0.0001 probability level. Non-signigicant difference was recorded between control and AHE alone group (One way ANOVA followed by Tukey’s multiple comparison)

### Effect of AHE on enzymatic antioxidants and oxidative stress markers in testis

Development of testicular impairment followed by CP exposure is mainly ascribed to free radical intervened oxidative distress and attenuation of antioxidant defense system. The effect of AHE treatments against CP intoxication of the antioxidant enzyme system are depicted in Tables 3 and 4. SOD (phase I antioxidant enzyme) causes dismutation of superoxide radicals to hydrogen peroxide (H_2_O_2_), while CAT and POD transform H_2_O_2_ to water and defend the cell from oxidative injury. Our results indicated the ability of CP to cause significant impairment on testicular tissue by decreasing the tissue protein content as well as CAT, POD, SOD and QR levels in addition to increasing the lipid peroxidation (TBARS) and H_2_O_2_ contents as opposed to control group (Tables 2 and 4).

**Table 3:** Effect of cisplatin (CP) and different treatments of AHE on testicular tissue antioxidant enzymes

Group	POD (U/min)	SOD (U/mg protein)	CAT (U/min)	QR (nM/min/mg protein)
Control	14.95 ± 0.34	1.413 ± 0.02	17.35 ± 0.16	141.7 ± 0.87
CP	8.92 ± 0.35^***^	0.787 ± 0.03^***^	10.48 ± 0.153^***^	94.26 ± 0.78^***^
AHE alone	15.13 ± 0.29^+++^	1.402 ± 0.014^+++^	17.95 ± 0.167^+++^	142.3 ± 0.754^+++^
CP + AHE	10.68 ± 0.37^***,++,###^	1.012 ± 0.031^***,++,###^	12.24 ± 0.152^***,+++,###^	101.9 ± 0.909^***,+++,###^
AHE + CP	13.42 ± 0.22^*,+++^	1.377 ± 0.061^+++^	16.91 ± 0.194^+++^	137.6 ± 0.974^*,+++^
CP + Sily	13.504 ± 0.23^*,+++^	1.321 ± 0.026^+++^	16.97 ± 0.20^+++^	137.4 ± 0.755^*.+++^

*n* = 6/group; values are expressed as mean ± SEM; ^*, **, ***^ indicate significance from the control group at *p* < 0.05, *p* < 0.001 and *p* < 0.0001 probability level, respectively; ^++, +++^ indicate significance from the CP group at *p* < 0.001and *p* < 0.0001 probability level, respectively; ### indicates comparison of AHE + CP pre-treated group vs. CP + AHE post treated group at *p* < 0.0001 probability level. Non-significant difference (*p* > 0.05) was recorded between control and AHE alone treated group in all parameters. (One way ANOVA followed by Tukeys multiple comparison tests)

**Table 4:** Effect of cisplatin (CP) and different treatments of AHE on testicular tissue antioxidant enzymes and GSH profile

Group	GSH (μM/g tissue)	GR (nM/min/mg protein)	GST (nM/min/mg protein)	γ-GT (nM/min/mg Protein)	GPx (nM/min/mg Protein)
Control	17.84 ± 0.77	158.4 ± 1.09	133.1 ± 0.95	326.5 ± 0.44	131.5 ± 1.72
CP	8.48 ± 0.415^***^	110.7 ± 1.29^***^	96.14 ± 0.84^***^	106.9 ± 0.59^***^	69.42 ± 1.62^***^
AHE alone	17.87 ± 0.54^+++^	159.6 ± 1.19^+++^	133.4 ± 1.25^+++^	329.4 ± 0.90^+++^	132.2 ± 0.98^+++^
CP + AHE	11.24 ± 0.65^***,+,###^	121.5 ± 1.36^***,+++,###^	111.7 ± 0.98^***,+++,###^	156.4 ± 0.49^***,+++,###^	83.91 ± 1.06^***,+++,###^
AHE + CP	16.06 ± 0.30^+++^	150.1 ± 1.14^**,+++^	128.4 ± 0.92^*,+++^	309.1 ± 0.89^***,+++^	126.2 ± 0.91^+++^
CP + Sily	16.15 ± 0.50^+++^	149.2 ± 1.72^**,+++^	128.2 ± 0.83^*,+++^	312.5 ± 1.03^***,+++^	126.3 ± 1.25^+++^

*n* = 6/group; values are expressed as mean ± SEM; ^*, **, ***^ indicate significance from the control group at *p* < 0.05, *p* < 0.001 and *p* < 0.0001 probability level, respectively; ^+++^ indicate significance from the CP group at *p* < 0.0001 probability level; ### indicates comparison of AHE + CP pre-treated group vs. CP + AHE post-treated group at *p* < 0.0001 probability level. Non-significant difference (*p* > 0.05) was recorded between control and AHE alone treated group in all parameters (One way ANOVA followed by Tukey’s multiple comparison tests)

CP + AHE and AHE + CP treatments significantly augment the suppressed protein content and antioxidant enzyme activity while decreasing the oxidative stress markers in contrast to CP alone treated group (Tables 3, 4 and 5). AHE + CP more efficiently (*p* < 0.001) prevented the CP deleterious effects as compared to CP + AHE treatment group.

**Table 5:** Effect of cisplatin (CP) and different treatments of AHE on testicular tissue protein, and oxidative stress markers

Group	Protein (μg/mg Tissue)	H_2_O_2_ (nM/min/mg Tissue)	Nitrite content (NO μM/ml)	MDA (nM/min/mg protein)
Control	1.558 ± 0.01	2.408 ± 0.09	41.97 ± 0.52	3.031 ± 0.12
CP	0.944 ± 0.04^***^	5.825 ± 0.06^***^	80.05 ± 0.62^***^	9.713 ± 0.18^***^
AHE alone	1.561 ± 0.05^+++^	2.349 ± 0.02^+++^	40.92 ± 0.37^+++^	2.95 ± 0.15^+++^
CP + AHE	1.352 ± 0.05^***,+++,###^	4.568 ± 0.02^***,+++,###^	62.77 ± 0.63^***,+++,###^	6.015 ± 0.15^***,+++,###^
AHE + CP	1.516 ± 0.03^+++^	3.216 ± 0.01^***,+++^	44.80 ± 0.48^*,+++^	3.87 ± 0.14^**,###^
CP + Sily	1.540 ± 0.02^+++^	3.145 ± 0.01^***,+++^	44.23 ± 0.76^+++^	4.058 ± 0.14^**,###^

*n* = 6/group; values are expressed as mean ± SEM; ^*, **, ***^ indicate significance from the control group at *p* < 0.05, *p* < 0.001 and *p* < 0.0001 probability level, respectively; ^++, +++^ indicate significance from the CP group at *p* < 0.001 and *p* < 0.0001 probability level, respectively; ### indicates comparison of AHE + CP pre-treated group vs. CP + AHE post-treated group at *p* < 0.0001 probability level. Non-significant difference (*p* > 0.05) was recorded between control and AHE alone treated group in all parameters (One way ANOVA followed by Tukey’s multiple comparison tests)

The effects of AHE treatment on phase II antioxidant enzymes like GSH, GR, GST, γ-GT and GPx, of testicular tissues are displayed in Table 3. CP administration extensively (*p* < 0.0001) decreased the glutathione status of GSH and the activity level of GR, GST, γ-GT and GPx while amplifing oxidative stress markers like NO, H_2_O_2_ and TBARS content (Table 4) in comparison to that of control group. In AHE + CP group GSH and GPx levels completely recovered to that in the control group. AHE + CP resulted in more significant (*p* < 0.001) improvement as compared to CP + AHE group, indicative of the protective effect of AHE against CP induced deteriorations. The effects of AHE + CP on testicular antioxidant enzymes were comparable to that of CP + silymarin treated group. Moreover, AHE alone presented nonsignificant changes in the activity level of testis antioxidant enzymes, protein content, oxidative stress markers and lipid peroxidation when compared to control group, indicating the nontoxic effect of the plant fraction.

### Effect of AHE treatment on testicular histology and morphometry

**Fig. 3:**
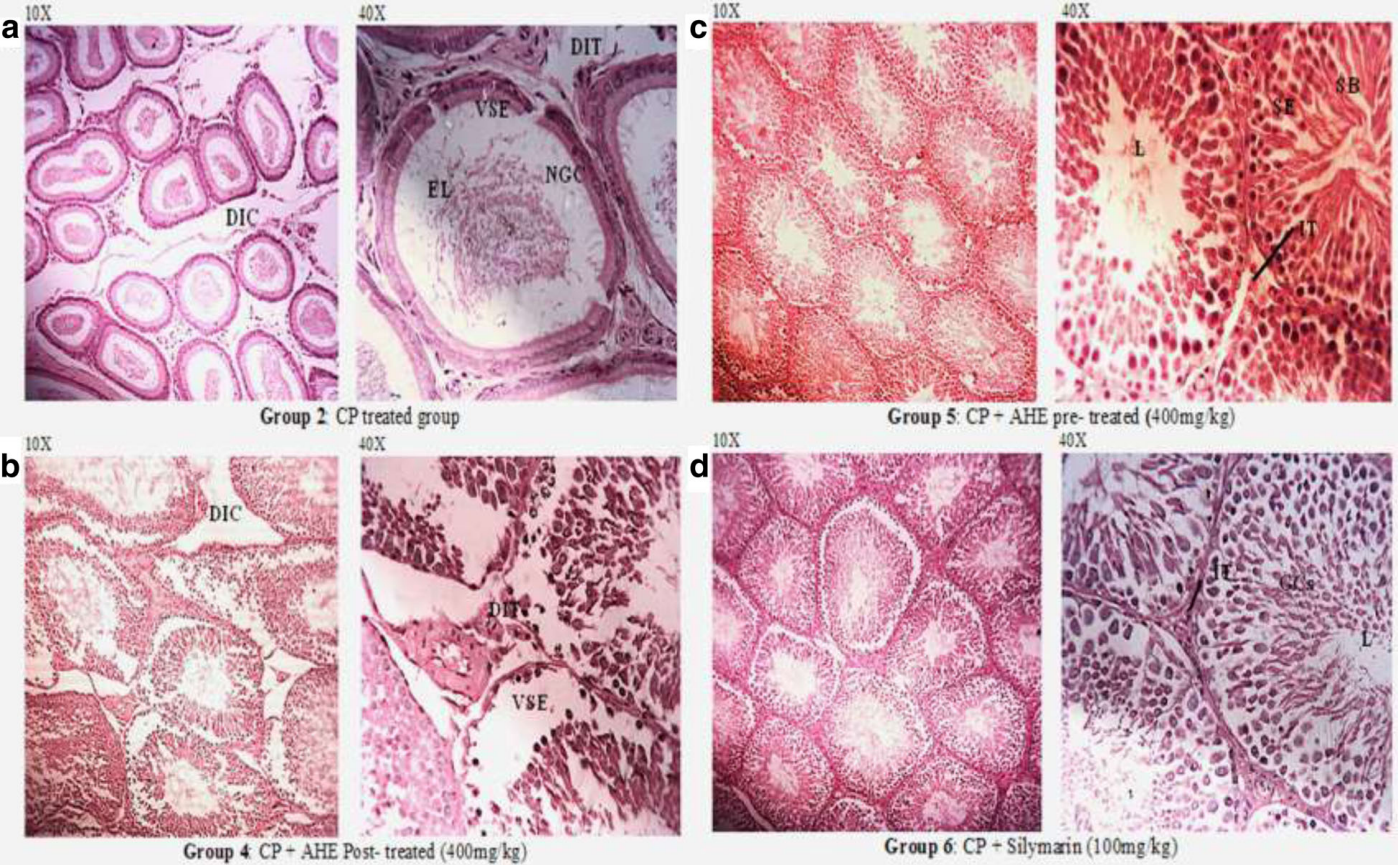
Microphotograph of rat testis (H&E stain) (**a**) CP group showing marked degenerative changes with severe germ cell aplasia, tubules with empty lumen, sloughing of the epithelial layer and degenerated interstitial space (**b**) CP + AHE group showing seminiferous tubule with elongating spermatids with a little degeneration in interstitial space and less sloughing in epithelial layer of cells (**c**) AHE + CP group showing filled lumen and compact seminiferous tubules with less interstitial space (**d**) Silymarin + CP group. AHE- *A. hydaspica* ethyl acetate extract, CP-Cisplatin, L-Lumen, SE-Seminiferous epithelium, ST-Seminiferous tubules, SB-Sperm bundles, Ld-Leydig cells, ST-Seminiferous tubules, SB-Sperm bundles, Ld-Leydig cells, VSE-vacuolated seminiferous epithelium, GCs- Germ cells, IT- Interstitial tissue, DST-distended seminiferous tubules, DIT-Disrupted interstitial tissue, DSE- disorganized seminiferous epithelium, EL-Empty lumen, DIC-Displaced interstitial cells, NGC-No germ cells

Figure 3 shows the histological examination of testicular tissues from different treated groups. Slides were studied at 10X and 40X magnifications. At 40X concentration, morphology and the stages of germ cells were observed while at 10X, spaces between the seminiferous tubules were examined between different treatment groups. Histopathological findings revealed the normal organization of germinal and Sertoli cells and active spermatogenesis in the seminiferous tubules without any histopathological injuries in control and AHE alone group (Fig. 1). CP inoculated animals presented inexorable testicular atrophy with austere cellular disorganizations, degenerations in seminiferous tubules and interstitium, and decreased thickness of germinal epithelium. CP inoculation also impelled the diminution of Leydig cells amongst the tubules and attenuated several stages in spermatogenesis. Disintegrated Sertoli cells were also noticed in the lumen. These alterations were markedly reduced with oral administration of AHE. Post treatment showed a protective effect in comparison to the CP group, yet maximum protection was seen in pretreatment group. Rats pre-treated with AHE and CP + silymarin displayed normal testicular morphology with trivial irregularities in germ cells arrangements and minor disintegration of seminiferous epithelium, moreover cell in various stages were observable showing active spermatogenesis. Current data showed that pre-treatment AHE extract offers more protection against the histopathological lesions impelled by CP in comparison to its administration after CP dose.

The seminiferous tubule diameter and epithelial height were reduced (*p* < 0.001) in the CP alone treated group when compared to the control group. However, in AHE + CP treated animals, tubular diameter and epithelial height showed significant increase (*p* < 0.0001) as compared to the CP alone treated group (Table 6). CP + AHE group showed significant (*p* < 0.001) improvement in seminiferous tubule diameter, but no effect on epithelial height was observed in comparison to CP group. Tubular lumen was significantly (*p* < 0.0001) increased in CP group compared to control, while AHE in both treatments significantly reduced the wide tubule lumen. Similarly, the area of the seminiferous tubule/unit area (%) was significantly decreased in the CP treated group as compared to the control group. A significant increase in the seminiferous tubule area was found in the CP + AHE and AHE + CP treated groups (*p* < 0.0001) when compared to the CP alone treated group (Table 5).The interstitial space (%) in unit area was significantly increased (*p* < 0.0001) in the CP treated group as compared to the control group. A significant reduction in interstitial space (%) was observed in the both CP + AHE and AHE + CP groups when compared with the CP treated group (*p* < 0.0001) (Table 6). Histopathological findings reinforced that *A. hydaspica* possesses the protective potential against CP-induced testicular toxicity and these results are in agreement of other investigated parameters.

**Table 6:** Effect of AHE treatment on testicular morphometric parameters in different experimental groups

Groups (*n* = 6)	Seminiferous tubule diameter (μm)	Seminiferous tubule epithelial height (μm)	Tubular lumen (μm)	Seminiferous tubule area (%)	Interstitial space (%)
Control	177.4 ± 2.04	70.90 ± 1.25	9.89 ± 0.63	86.29 ± 1.05	13.7 ± 1.05
CP	163.9 ± 1.60^***^	21.15 ± 0.86^***^	66.48 ± 0.61^***^	45.44 ± 0.64^***^	54.56 ± 0.64^***^
AHE alone	173.7 ± 1.54^+++^	71.52 ± 0.99^+++^	9.99 ± 0.65^+++^	86.69 ± 0.60^+++^	13.31 ± 0.54^+++^
CP + AHE	168.8 ± 1.24^**,++,##^	24.18 ± 1.09^***,###^	63.27 ± 0.72^***,+,###^	69.33 ± 0.41^***,+++,###^	30.67 ± 0.42^***,+++,###^
AHE + CP	178.3 ± 1.62^+++^	66.03 ± 0.68^*,+++^	11.18 ± 0.60^+++^	82.22 ± 0.60^**,+++^	17.78 ± 0.60^**,+++^
CP + Sily	173.8 ± 1.25^+++^	66.33 ± 0.79^*,+++^	11.43 ± 0.47^+++^	82.44 ± 0.35699^**,+++^	17.56 ± 0.35^**,++^

*n* = 6/group; data presented as mean ± SEM;*** indicate significance from control group at *p* < 0.0001 probability level, +, +++ indicate significance from CP group at *p* < 0.05 and *p* < 0.0001 probability level, ^##^, ^###^ indicate significance from AHE + CP group at *p* < 0.001 and *p* < 0.0001 probability level respectively

### Effect of AHE treatment on cisplatin induced DNA damage

**Fig. 4:**
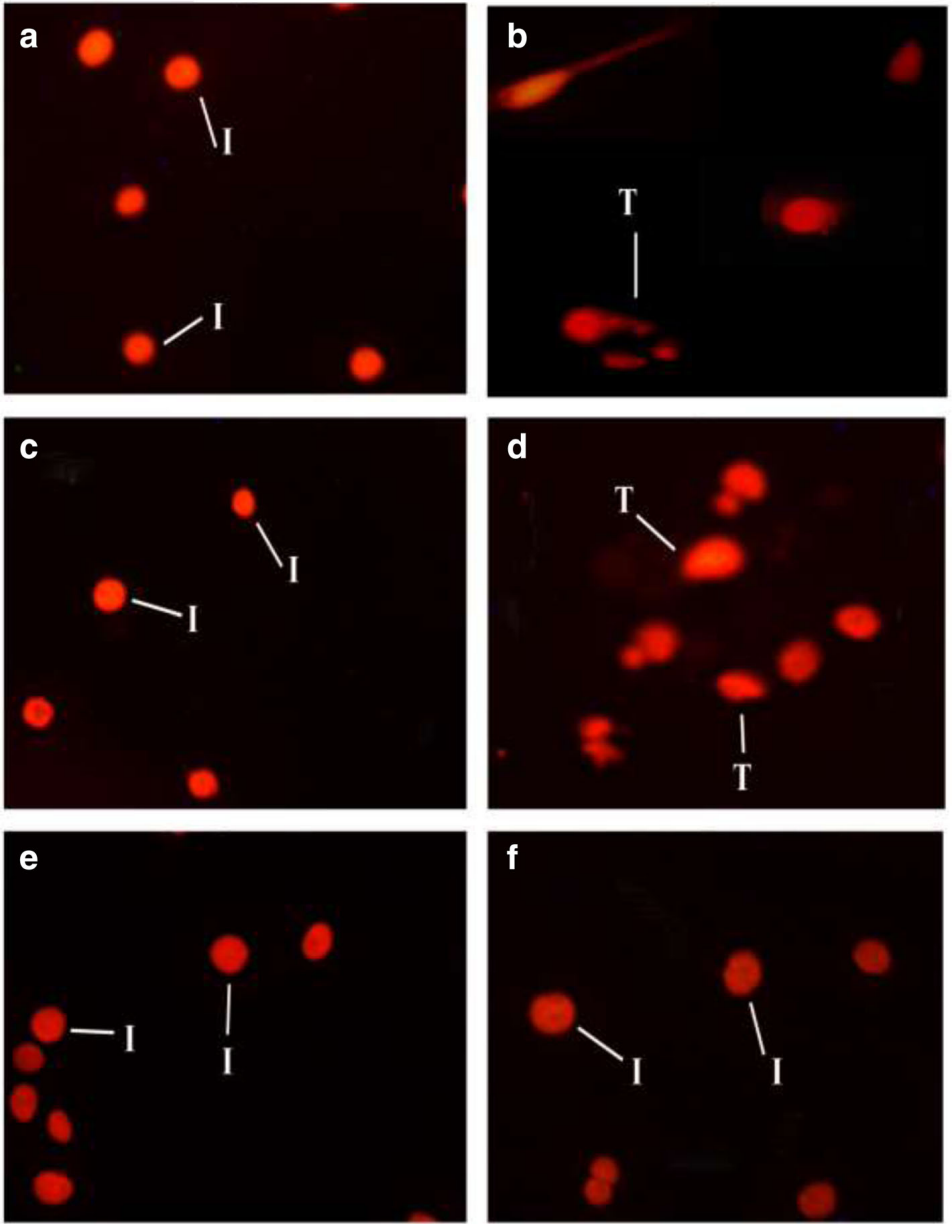
Fluorescence photomicrograph of testicular cells revealing protective effect of AHE against CP induced genotoxicity. **a** Control group, **b** CP (7.5 ml/kg b.w, i.p), **c** AHE alone, **d** CP + AHE, **e** AHE + CP,**f** Silymarin + CP. I: Intact Comet head; T, Comet tail

**Fig. 5:**
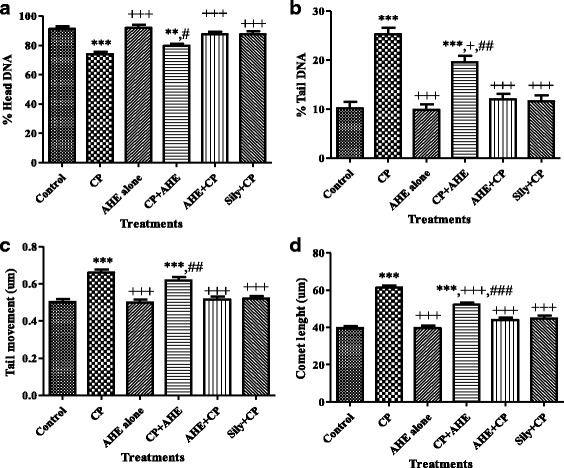
Comet scoring parameters indicating the protective effect of AHE against CP induced DNA damages. **a** Percent head DNA, **b** Percent tail DNA, **c** Tail movement (μm), **d** Comet lengh (μm). *n* = 6/group; data presented as mean ± SEM;**, *** indicate significance from control group at *p* < 0.001 and *p* < 0.0001 probability level, +, +++ indicate significance from CP group at *p* < 0.05 and *p* < 0.0001 probability level, ^##^, ^###^ indicate significance from AHE + CP group at *p* < 0.001 and *p* < 0.0001 probability level respectively

Figure 4 depicts microphotograph of different treatment groups and protective potential of AHE on genotoxicity. Maximum DNA damage was obsereved in CP treated group (4b). Figure 5 depicts the quantitative analysis of different comet scoring parameters studied. Greater proportion of cells with intact DNA (5a), less concentration of tail DNA (5b), reduced tail movement (5c) and an insignificant number of comets with very tiny tail length (5d) were observed in control group. CP inoculation induced DNA demage and resulted in significant (*P* < 0.0001) increase in tail DNA concentration (Fig. 5b), tail movement (Fig. 5c) and comet length (Fig. 5d) while marked (*P* < 0.0001) decrease in concentration of DNA in comet head was recorded (Fig. 5a). Treatment with AHE ameliorated the CP-induced DNA damages and the comet values were restored towards the control level (Fig. 5). AHE + CP reduced the DNA damages more convincingly (*p* < 0.001) as compared to CP + AHE treated group, specifying the preventing potency of AHE. Concentration of DNA in head was sharply enhanced in head of comet along with significant (P < 0.0001) decrease in tail moment with the AHE pretreatment was noticed.Treatment of AHE + CP showed equal potency as CP + silymarin in protecting the testicular DNA damages and restored the comet values towards the control group. AHE administration to rats resulted in non-significant differences in comet parameters compared to control group.

## Discussion

Male germ cells are known to be one of the tissues prone to CP toxicity. CP treatment, causes testicular toxicity by inducing oxidative stress, spermatotoxicity and DNA damage; which results infertility-causing complications and hence, the protection of testicular tissue remains a critical issue whenever CP is employed in cancer chemotherapy [4, 10, 53, 54]. The therapeutic potential of medicinal plants is accredited to their secondary metabolites. The scavenging of free radicals by the plant derived product may offer a natural alternative method to tackle oxidative stress persuaded tissue damages. Polyphenolic compounds play an important role in the prevention of CP induced oxidative trauma and testicular injuries [1].

In the present study, we measured several biochemicals, hormonal and histological parameters related to testicular toxicity and oxidative stress in the testis tissue to evaluate the protective effect of AHE against CP-induced reproductive toxicity in male rats.

Our findings on CP induced testicular impairment endorsed the previous findings that CP treatment causes biochemical and testicular tissue alterations such as in germinal epithelium and long term failure of spermatogenesis due to its alkylation property [9, 53, 55]. The earlier work of Turk et al. also demonstrated that CP decreased the testicular weight, epididymis and seminal vesicle in treated rats [56]. However, different mechanisms have been anticipated on the damaging aspect of the drug. A strategy to decrease the prevalence of severe lateral-influences of anticancer drugs with conservation of its chemotherapeutic usefulness is indispensable.

Serum gonadotropin releasing hormone (GnRH) including LH, FSH and T levels aid in making conclusions regarding reproductive pathologies. The fall in serum T concentration describes either direct effect of the chemical at Leydig cell level or an indirect action via alteration in hormonal regulation of hypothalamic-pituitary axis (HPA) due to oxidative stress in drug treated animals [57]. It was also reported that abnormal concentration of intratesticular testosterone inhibit spermatogenesis [58]. The production of testosterone in Leydig cells is stimulated by LH, which activates FSH to bind with Sertoli cells to stimulate spermatogenesis [33]. The outcome of current investigation with reference to the testicular function marker enzymes in serum, revealed that CP exhibited a noteworthy suppression of T, LH and FSH concentrations. The suppression of T by CP has been reported previously [59], however, the effect of CP on LH and FSH in our investigation is in contradiction as they reported that CP (7.5 mg/kg, iv) administration did not alter serum LH or FSH while it decrease serum and testicular testosterone [60]. Garcia et al. also reported that the inhibitory potential of CP on testosterone synthesis was due to ROS [61, 62]. Administration of AHE results in potential intensification of lowered levels of T, LH and FSH and might result in regulation of HPA. The decrease in serum testosterone might be consequent of direct toxic effect of CP on the Leydig cells, as previous study revealed that antineoplastic agents can disrupt Leydig cells directly [63]. Steroidogenesis in the male rats is triggered by GnRH, which elicit the production and release of LH, which then binds to LH receptors on the membrane of Leydig cells to upregulate testosterone production [64]. The reduction in serum LH level observed in current study possibly is the outcome of impairment in the negative feedback mechanism of hypothalamic-pituitary axis [65]. AHE + CP, more significantly maintained serum hormone levels compared to CP + AHE, indicating the preventive potential of AHE. Furthermore, reduction in the weight of the testes in CP treated rats revealed the reduced availability of androgens [66].

Oxidative stress, a situation of disproportion between the free radicals and antioxidant defense system, is an imperative cause in the pathogenesis of various ailments [67]. The level of antioxidant enzymes, including SOD, POD, CAT, QR as well as GSH, GPx, GR, GST and gamma GT were assessed to determine the potential protective influence of AHE against CP induced oxidative stress mediated gonadotoxicity in mature male rats. Furthermore, TBARS as a marker of lipid peroxidation and H_2_O_2_ and NO levels as a marker of oxidative and nitrosative stress were estimated in testicular tissue. Previous studies authenticated the prominence of oxidative stress in the pathogenesis of CP mediated gonadotoxicity [54, 66]. The unnecessary concentration of ROS in the cisplatin treated rats makes spermatozoa extremely liable to impairment due to the high content of polyunsaturated fatty acids in their plasma membrane [68]. Moreover, Sharma and Agarwal reported that enhanced lipid peroxidation in CP treated rats, destroys the structure of spermatozoa accompanied by the loss of its motility and impairment of spermatogenesis [69]. The AHE + CP as well as CP + AHE treatment strategies produced a potential increase in depleted antioxidant enzyme levels in the welfare of oxidative trauma in vivo. AHE treatment significantly ameliorated the increased levels of oxidative stress markers, i.e. TBARS, H_2_O_2_ and NO, and pre-treatment restored the levels near normal. The antioxidant enzyme levels showed noticeable increase while oxidative stress marker levels were significantly diminished in AHE + CP group in comparison with CP + AHE. The mechanism behind AHE protective antioxidant effect might be due to its flavonoids enrichment; as flavonoids from plants and fruits were potent OH•, O_2_• − scavengers, chelate metal ions and exert a synergistic effect with other antioxidant metabolites [58]. We strongly beleive that polyphenolic compounds of AHE might be responsible for the preventing CP induced testicular impairments.

The testicular protein level showed a significant decrease in CP treated groups compared to control. During abnormal spermatogenesis, the testicular proteins become demolished [70]. The highly significant reduction in protein content in CP treated group may be due to defective spermatogenesis.

Testicular histomorphology of CP treated groups revealed severe spermatogenesis impairment, increase in germ cell death and testicular atrophy, causing degenerative vicissitudes in germinal epithelium and vacuolated spermatognial cells. Depletion of Leydig cells and degeneration of Sertoli cells were also noticed in the CP treated group. Our findings are in agreement with Amin et al. [71] who reported that degenerative changes in CP treated groups has an association with increase oxidative stress, DNA damage and decrease antioxidant defense system. The structural alterations observed in the current experiments may be corroborated with reductions in serum testosterone levels in respective groups. Giri and his colleagues revealed that treatment of CP reinforces ROS production inside the cell and hence sperms were in exposure to oxidative damage and Vitamin C ameliorates this damaging effect of CP [72]. Previous studies also reported that chemotherapy consequently decrease the number of Sertoli cells [73, 74]. In the present work, low serum levels of testosterone, LH and FSH and increase in H_2_O_2_ and other oxidative markers in the testicular tissue corroborate with the observations of structural alterations and inhibition of the spermatogenesis in CP injected groups. Overall, it was observed that considerable germ cell damage instigated by anticancer drugs is proceeded by a sharp degenerations in testicular histological parameters [75]. Treatments with AHE significantly protect the testicular morphology; pre-treatment outcomes in the marked prevention of CP induced toxicity. The mechanism of AHE to protect testicular damage is through its antioxidant and free radical scavenging potential due to the presence of polyphenols and flavonoids [24, 25].

Comet assay was performed to estimate the DNA damages incited with CP generated ROS in testicular tissue. In current study significant increase in tail moment, comet length, % DNA in the tail was recorded in CP group. The oral dose significantly ameliorates the altered comet parameters and the DNA protective effect was more pronounced in AHE + CP group supporting the previous observations that AHE has preventive potential. Similar results were reported by Sahreen et al. [12] where genotoxicity was induced by CCl_4_ in renal tissues and ameliorated with extract of *Carissa opaca* fruit. Our results suggested that *A. hydaspica* is a worthy candidate to inhibit the DNA damage in testicular tissues.

**Fig. 6:**
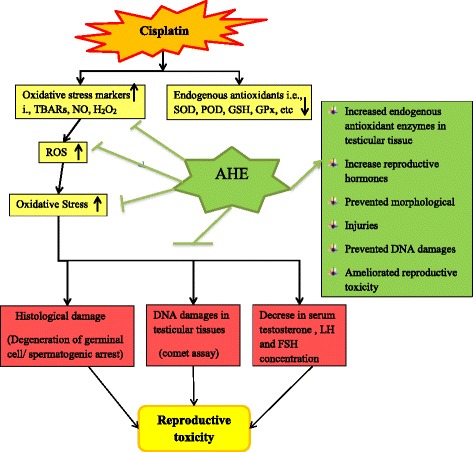
Hypothetical pathway describing the possible underlying mechanism of CP induced reproductive toxicity and protective effect of AHE

Figure 6 summarizes the possible mechanism of AHE protective effect against CP mediated reproductive toxicity. The treatment of AHE for CP induced testicular toxicity has efficiently ameliorated the altered level of antioxidant enzymes, biochemical markers, DNA damages and histoarchitecture of testis in a rat model. Similarly the derailed level of hormones in the serum was reverted towards the level of control with AHE in the CP treated rats. The protective and curative activities manifested by the AHE in this investigation are encouraging as the more efficacious therapeutics is needed to have multitude role in the oxidative stress related disorders. These studies would be helpful in the designing of biologically active drugs having minimum side effects and cost-effective.

## Conclusion

In conclusion, the current investigation ratifies that reproductive toxicity induced by CP is related to increased oxidative stress. AHE as a potential antioxidant might be utilized in conjuction with or before chemotherapeutic drug administration to avoid the associated side effects. Results clearly augment the defensive mechanism of AHE against oxidative stress induced by CP and provide confirmation about its therapeutic use in reproductive abnormalities.

## Abbriviations

AHE: *A. hydaspica* ethyl acetate extract.
CP: Cisplatin
FSH: Follicle stimulating hormone
GnRH: Gonadotrophin releasing hormone
Gpx: Glutathione peroxidase
GSH: Reduce glutathione
HPA: Hypothalamo-pituitary axis
LH: Luteinizing hormone
O_2_•−: Superoxide
POD: Peroxidase
SOD: Superoxide dismutase
TBARs: Thiobarbituric acid reactive substances
